# Rice Protein Extracted by Different Methods Affects Cholesterol Metabolism in Rats Due to Its Lower Digestibility

**DOI:** 10.3390/ijms12117594

**Published:** 2011-11-07

**Authors:** Lin Yang, Jiahou Chen, Tong Xu, Wei Qiu, Yan Zhang, Lanwei Zhang, Fuping Xu, Hongbo Liu

**Affiliations:** 1 School of Food Science and Engineering, Harbin Institute of Technology, 73 Huanghe Road, Harbin 150090, China; E-Mails: lanweizhang@yahoo.com.cn (L.Z.); chinacentre@163.com (F.X.); 2 School of Chemical Engineering and Technology, Harbin Institute of Technology, Harbin 150001, China; 3 Heilongjiang Provincial Environmental Monitoring Central Station, Harbin 150056, China; E-Mail: cjh6617@126.com; 4 Heilongjiang Provincial Hospital, Harbin 150036, China; E-Mail: furukawa1964@yahoo.com.cn; 5 State Key Laboratory of Urban Water Resources and Environment, Harbin Institute of Technology, Harbin 150090, China; E-mail: qiuwei@hit.edu.cn; 6 School of Municipal and Environmental Engineering, Harbin Institute of Technology, Harbin 150090, China; 7 College of Bioinformatics Science and Technology, Harbin Medical University, Harbin 150081, China; E-Mails: yangyou1225@yahoo.com.cn (Y.Z.); hongbo919@gmai.com (H.L.)

**Keywords:** rice protein, digestibility, cholesterol, absorption, rats

## Abstract

To elucidate whether the digestibility is responsible for the hypocholesterolemic action of rice protein, the effects of rice proteins extracted by alkali (RP-A) and α-amylase (RP-E) on cholesterol metabolism were investigated in 7-week-old male Wistar rats fed cholesterol-free diets for 3 weeks. The *in vitro* and *in vivo* digestibility was significantly reduced by RP-A and RP-E as compared to casein (CAS). The digestibility was lower in RP-E than that of RP-A. Compared with CAS, the significant cholesterol-lowering effects were observed in rats fed by RP-A and RP-E. Fecal excretion of bile acids was significantly stimulated by RP-E, but not by RP-A. The apparent cholesterol absorption was more effectively inhibited by RP-E than RP-A because more fecal neutral sterols were excreted in rats fed RP-E. There was a significant correlation between protein digestibility and cholesterol absorption (*r* = 0.8662, *P* < 0.01), resulting in a significant correlation between protein digestibility and plasma cholesterol level (*r* = 0.7357, *P* < 0.01) in this study. The present study demonstrates that the digestibility of rice protein affected by extraction method plays a major role in the modulation of cholesterol metabolism. Results suggest that the hypocholesterolemic action induced by rice protein with lower digestibility primarily contribute to the inhibition of cholesterol absorption.

## 1. Introduction

Rice is a staple cereal and widely consumed in the world. Recent attempts have been focused on the physiological functions of rice to prevent the life style-related diseases [1–4], in which the association of rice protein (RP) consumption with modulation of plasma cholesterol level has been demonstrated in some studies [5–9]. However, evidence as to how the hypocholesterolemic mechanism exerted by rice protein works is lacking.

As for isolation of rice protein, alkaline treatment and α-amylase degradation are two major industrial processing [5,10]. Alkaline treatment is a common process for rice protein extraction, by which the digestibility of rice protein is suggested to be effectively improved rather than by α-amylase degradation [5,11,12]. Morita *et al.* indicated that the true digestibility of rice protein extracted by alkaline treatment was higher than that treated by α-amylase, and showed that rice protein isolates produced by the two different methods (alkaline treatment and α-amylase degradation) could lower serum cholesterol concentration in Sprague-Dawley rats compared with casein [5]. Taken together, these findings provided evidence that there may be a link of extraction method and protein digestibility which may be connected to the physiological function of rice protein.

Some studies have suggested that the biological utilization of a protein is primarily dependent on its digestibility by gastric, pancreatic and intestinal peptidases [13–15], further providing the insight that the digestibility may play a major role in the modulation of physiological function of dietary protein. In addition, the most frequently suggested mechanism responsible for the cholesterol-lowering effect of plant protein is the interference with enterohepatic circulation of cholesterol, leading to an inhibition of intestinal cholesterol absorption and an increase in fecal steroid excretion [16]. Taking advantage of this view, some studies suggest that the low digestibility of dietary protein is a factor involved with the inhibition of intestinal cholesterol absorption and the disturbance of enterohepatic circulation to lead to the cholesterol-lowering action [17,18]. Thus, the digestibility of rice protein may be a major factor to influence cholesterol metabolism.

Therefore, in the present study, two extraction methods for preparation of rice protein, namely, an alkaline treatment and heat-stable α-amylase degradation, were developed to evaluate and compare the physiological function of rice protein. The key question addressed are: (1) whether the digestibility is responsible for the hypocholesterolemic action of rice protein and (2) how the digestibility of rice protein possesses a vital function in improving cholesterol metabolism in growing rats fed cholesterol-free diets. In addition, the present work also focused on the effect of rice protein on triglyceride metabolism.

## 2. Experimental Section

### 2.1. Protein Sources

Casein (CAS) (Gansu Hualing Industrial Group, Gansu, China) and rice proteins extracted from *Oryza sativa* L. cv. *Longjing* 26 (Rice Research Institute of Heilongjiang Academy of Agricultural Sciences, Jiamusi, China) were used as the dietary protein sources. Two methods were conducted for preparation of rice proteins, a classical extraction method with alkaline followed by precipitation with acidic solution (RP-A) [5], and a method for rice protein isolation by starch degradation using a heat-stable α-amylase (Sigma, St. Louis, MO, USA) (RP-E) [5].

The chemical composition of dietary proteins was analyzed, according to the previous study [6]. Amino acid analysis of these proteins was performed using a Biochrom 30 amino acid analyzer (Biochrom, Holliston, MA, USA) according to Yang *et al*. [6].

### 2.2. In Vitro Digestion Study

The lipid fraction of protein samples (CAS, RP-A, RP-E) was removed by chloroform-methanol (2:1, v/v) extraction [19], and the *in vitro* digestion of CAS, RP-A and RP-E with pepsin and pancreatin was performed according to previous study [17].

Of the defatted sample (CAS, RP-A, RP-E), protein solution (5% w/v, in distilled water) was adjusted to pH 2.0 with dilute HCl, and incubated at 37 °C with 5 mg of porcine pepsin (Sigma, St. Louis, MO, USA). During the pepsin digestion, the digest was sampled at intervals of 10 min, 30 min, 45 min, 1 h and 2 h. After deproteinization by 30% trichloroacetic acid, free amino group, which was reacted with 2,4,6-trinitrobenzenesulfonic acid at 37 °C for 2 h, was measured at 420 nm to evaluate the pepsin digestion.

The *in vitro* protein digestibility was conducted according to the method of Mohamed *et al*. and Ali *et al*. [13,20], with slight modification. After 2 h of pepsin digestion, the peptic digest was adjusted to pH 8.5 with NaHCO_3_, and then treated with 15 mg porcine pancreatin (Sigma, St. Louis, MO, USA). The mixture was incubated at 37 °C for 24 h. At appropriate intervals (4, 6, 8 and 24 h), samples were taken and treated with 30% trichloroacetic acid and centrifuged at 12,000× *g* for 5 min at room temperature. After centrifugation, the acid-soluble fraction was estimated according to the method of Lowry *et al.* [21]. *In vitro* protein digestibility was calculated as: *In vitro* protein digestibility (%) = Protein in the supernatant/Protein in the sample × 100.

### 2.3. Animals and Diets

The present experiments followed the same as the previous study [6]. 7-week-old male Wistar rats were purchased from Animal Center of Harbin Medical University (SCXK20020002, Harbin, China) and individually housed in metabolic cages in a room maintained at 22 ± 2 °C under a 12 h light-dark cycle (07:00–19:00 for light). Rats were allowed free access to commercial pellets (Animal Center of Harbin Medical University, Harbin, China) for 3 days. After acclimatization, rats were randomly divided into three groups of similar body weight. Each group consisted of six rats.

All animals were fed *ad libitum* with experimental diets according to the formula recommended by American Institute of Nutrition [22]. For 3 weeks, growing rats were fed cholesterol-free diets with dietary protein level of 20% (as crude protein) of CAS, RP-A and RP-E, respectively. Diets were completed to 100% with starch. The composition of experimental diets is shown in Table 1.

### 2.4. Samples Collection

During the feeding period, food consumption and body weight were recorded daily in the morning before replenishing the diet. Feces were collected for the final 3 days of the experimental period and dried to a constant weight and ground to a fine powder for fecal steroids determination according to Yang *et al*. [6].

At the end of the feeding period, the rats were deprived for 18 h and then sacrificed. Blood was withdrawn from abdominal vein into a heparinized syringe under anesthesia with sodium pentobarbital (50 mg/kg body weight), immediately cooled on ice and separated by centrifugation at 12,000× *g* for 5 min. The plasma obtained was frozen at −20 °C until analysis. After blood collection, the liver, perirenal fat and epididymal fat were excised immediately, rinsed in saline and weighed after blotted on a filter paper. The whole liver was cut into three portions and quickly freeze-clamped in liquid nitrogen and stored at −80 °C until analysis.

### 2.5 Plasma Lipid Analysis

Plasma concentrations of total cholesterol (TC), low-density lipoprotein cholesterol (LDL-C), high-density lipoprotein cholesterol (HDL-C) and triglyceride (TG) were measured colorimetrically with commercial kits (Nanjing Jiancheng Bioengineering Institute, Nanjing, China). Plasma very-low-density lipoprotein cholesterol (VLDL-C) was calculated as: VLDL-C = TC − HDL-C − LDL-C.

### 2.6. Liver Lipid Analysis

The lipids in the liver were extracted and purified according to the method of Folch *et al.* [19], and were analyzed as described by Yang *et al.* [6]. Samples of liver were extracted with chloroform/methanol (2:1, v/v). Total- and free-cholesterol and triglyceride were measured with a commercial kit (Nanjing Jiancheng Bioengineering Institute, Nanjing, China). Total lipids were determined gravimetrically. The concentration of esterified cholesterol was calculated as: esterified cholesterol = total cholesterol − free cholesterol. Cholesterol esterification ratio was described as: cholesterol esterification ratio (%) = (total cholesterol − free cholesterol) × 100/total cholesterol.

### 2.7. Determination of Fecal Excretion

Fecal bile acid and neutral sterol concentrations were measured as described in detail previously [6]. Total bile acids were determined by 3α-hydroxysteroid dehydrogenase (Sigma, St. Louis, MO, USA) with sodium taurocholate as the standard. Cholesterol and coprostanol were identified by their retention time and mass-spectrum standard material by GCMS (GCMS QP5050A, Shimadzu, Kyoto, Japan), and the other sterols were identified by mass-spectrum and calculated by coprostanol’s standard curve. Apparent cholesterol absorption was calculated as: apparent cholesterol absorption = cholesterol intake − neutral sterol excretion. After lipid extraction by chloroform-methanol (2:1, v/v) [19], fecal triglyceride in the extracted lipid was assayed with a commercial kit (Nanjing Jiancheng Bioengineering Institute, Nanjing, China). Fecal nitrogen content was determined by Kjeldahl method [23]. Apparent digestibility of protein was calculated as: Apparent protein digestibility (%) = (Protein intake − Fecal protein) × 100/Protein intake.

### 2.8. Statistical Analysis

Data are presented as means ± SEM. Differences between groups were examined for statistical significance using the one-way analysis of variance (ANOVA), and then determined with the least significant difference test. The criterion for significance was *P* < 0.05.

## 3. Results

### 3.1. Chemical Composition and Amino Acids of Dietary Protein

Chemical composition of dietary protein was shown in Table 2. As the dry matter basis, the rice proteins used in the present study included a rather high CP (crude protein) content: RP-A, 95.8%; RP-E, 89.3%. Ash and lipid contents were almost negligible in all dietary proteins (CAS, 2.2%; RP-A, 1.8%; RP-E, 2.3%). As carbohydrate, including fiber, they were about 0.8% in CAS, 2.0% in RP-A and 7.2% in RP-E.

It is notable that the contents of fiber in RP-A and RP-E were higher than the contents of carbohydrate in them. To explain this phenomenon, the washing procedure, during which carbohydrate might be lost, should be taken into account in this study. Interestingly, the residual of fiber was higher in RP-E after α-amylase extraction than that in RP-A extracted by alkaline, implying alkaline treatment could reduce the fiber content in rice protein extract.

The amino acid compositions were shown in Table 3. As compared with CAS, rice proteins have higher levels of Asp, Glu, Gly, Ala, Cys and Arg, whereas they have lower content of Lys, Thr and Pro. As a result, the higher ratio of Arg/Lys was found in RP-A (2.56) and in RP-E (3.15) than that in CAS (0.44). Also, it should be noted in this study that the contents of Lys and Thr in RP-A and RP-E were lower than that in rice flour (Lys, 37.7; Thr, 31.97), suggesting that the first limiting amino acid (Lys) and the second limiting amino acid (Thr) of rice protein seem be easily lost during the extraction processing, despite the fact that the extraction method was different.

### 3.2. In Vitro Digestibility

**Figure 1:**
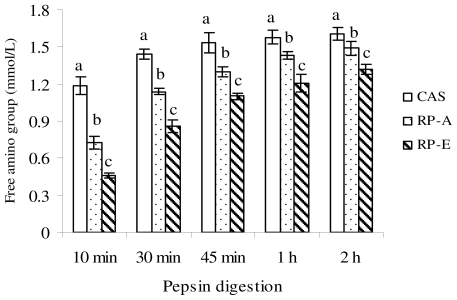
Effects of dietary proteins on free amino groups during pepsin digestion (10 min, 30 min, 45 min, 1 h, 2 h). Values are means ± SEM (*n* = 3). Bars for each value with different letters are significantly different (*P* < 0.05).

As illustrated in Figure 1, the *in vitro* digestibility with pepsin and pancreatin was significantly lower in RP-A and RP-E than that in CAS, in accordance with the findings investigated by Morita *et al*. [5,10].

The *in vitro* digestibility induced by pepsin was determined by the changes in free amino group during the 2 h of pepsin digestion (Figure 1). At any incubation time, concentrations of the free amino group were significantly lowered by RP-A (from 38.1% to 7.5%) and RP-E (from 61.0% to 18.0%) as compared with CAS (*P* < 0.05).

**Figure 2:**
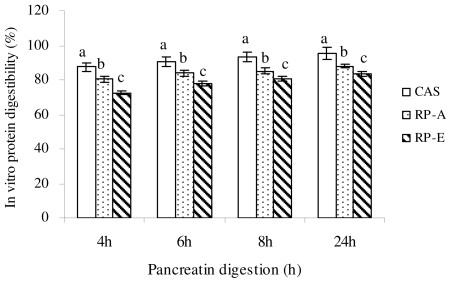
The *in vitro* digestibility of dietary protein after pepsin (2 h) and pancreatin (4, 6, 8, 24 h) digestion. Values are means ± SEM (*n* = 3). Bars for each value with different letters are significantly different (*P* < 0.05).

After 2 h pepsin and appropriate (4, 6, 8, and 24 h) pancreatin digestion, the *in vitro* digestibility of CAS, RP-A and RP-E was measured (Figure 2). With the similar tendency of pepsin digestion, the *in vitro* digestibility of RP-A and RP-E at any pancreatic incubation were all significantly lower than that of CAS (*P* < 0.05), indicating the lower *in vitro* digestibility was induced by RP as compared with CAS. Moreover, it must be noted that RP-E extracted by α-amylase exhibited a significant reduction in the *in vitro* digestibility, both pepsin digestion and pancreatin digestion, as compared with RP-A treated by alkali (*P* < 0.05). As a result, our results confirmed and supported the previous observation that the digestibility of RP could be improved by alkali treatment [11,12], suggesting that the *in vitro* digestibility of RP was closely associated with the extraction method.

### 3.3. Food Intake and Body Weight

Body weight gain of growing rats were significantly reduced in RP-A and RP-E groups (*P* < 0.05), as compared with CAS. Food intake was not significantly different among groups, suggesting that dietary protein did not affect food intake (Table 4).

### 3.4. Plasma Lipids and Lipoprotein Profiles

As shown in Table 4, plasma TC concentrations were significantly lowered in growing rats fed RP-A (TC: 12.5%) and RP-E (TC: 16.0%) as compared with CAS (*P* < 0.05).

Similarly, accompanying the decreased level of TC, plasma VLDL-C and LDL-C concentrations were also distinctly lower in growing rats fed RP-E and RP-A than those fed CAS (*P* < 0.05), whereas HDL-C level was not significantly different among experimental groups (*P* > 0.05). As a result, the ratio of non-HDL-C/HDL-C was significantly lowered by 29.7% and 34.4% in RP-A and RP-E, respectively, compared with CAS.

With the decreased plasma TC, plasma TG concentration was also lowered by RP feeding, as compared with CAS (Table 4). However, a marked reduction in plasma TG concentration was only produced by RP-E (*P* < 0.05). As for the ratio of TG to HDL-C, the decrease was found in RP-A and RP-E compared to CAS, but there was no significant difference of TG/HDL-C ratio among experimental groups (*P* > 0.05).

### 3.5 Hepatic Lipids and Deposit Fat

The liver weights of rats fed RP-A and RP-E were significantly lower than those fed CAS (*P* < 0.05). The liver weights in growing rats fed RP-E did not differ from those fed RP-A (*P* > 0.05) (Table 5).

As shown in Table 5, hepatic accumulations of total lipids, total cholesterol and triglyceride in RP-feeding groups were significantly lower than those in the CAS group (*P* < 0.05), in accordance with the previous study [6]. The hepatic cholesterol-lowering actions induced by RP-feeding were mainly reflected by diminished concentrations of free- and esterified cholesterol. Compared with CAS, RP-A and RP-E significantly reduced hepatic esterified cholesterol levels by 43.6% and 56.4%, respectively. These changes resulted in the significant reduction in hepatic cholesterol esterification ratio, which fell by 24.7% in RP-A and 36.9% in RP-E, as compared with CAS.

With the similar tendency of hepatic lipid accumulation, the deposits of perirenal fat and epididymal fat were also inhibited by RP-A (perirenal fat: 9.5%; epididymal fat: 9.5%) and RP-E (perirenal fat: 12.2%; epididymal fat: 12.3%), as compared with CAS.

### 3.6. Fecal Excretion

The 3-d fecal output was shown in Table 6. Compared with CAS, RP-E produced marked fecal output by increasing 9.0% (*P* < 0.05), while fecal output in RP-A did not differ from CAS (*P* > 0.05).

Compared with CAS, RP-E significantly enhanced fecal bile acids excretion by 35.1% (*P* < 0.05), whereas fecal bile acid excretion was not significantly affected by RP-A (*P* > 0.05). These findings were in accordance with our previous studies, in which rice protein extracted by alkaline treatment did not stimulate fecal bile acids excretion as comparison with CAS [6].

As shown in Table 6, fecal neutral sterol excretion was significantly stimulated with RP-feeding (*P* < 0.05). Compared with CAS, fecal neutral sterols excretion was 111.4% higher in growing rats fed RP-A, while RP-E increased 167.7%. Among the neutral sterols excreted, the concentrations of cholesterol and coprostanol were also significantly affected by RP-feeding, accounting for 85.8% and 131.8% enhancements of cholesterol and coprostanol in RP-A and RP-E, respectively, as compared with CAS (Table 6). These results clearly indicated that RP-E could produce more excretion of fecal sterols than those in RP-A group (*P* < 0.05).

As for fecal triglyceride excretion, the distinct increases were also observed in RP-A and RP-E as compared with CAS (*P* < 0.05) (Table 6). There was no difference of fecal triglyceride output was found in RP-E and RP-A (*P* > 0.05). As shown in Table 6, fecal excretions of nitrogen in the RP groups were significantly higher than that in the CAS group (*P* < 0.05), leading to the result that the apparent protein digestibility was significantly lower in RP-E and RP-A as compared with CAS (*P* < 0.05). This observation was in agreement with the results shown by Morita *et al*. [5,10]. Moreover, to support our *in vitro* finding that the digestibility of RP-E was significantly lower than RP-A (Figures 1 and 2), we also found that there was a marked difference of apparent protein digestibility between RP-E and RP-A (*P* < 0.05) (Table 6).

**Figure 3:**
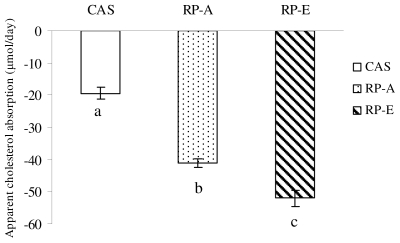
Apparent cholesterol absorption in growing rats fed cholesterol-free diets. Values are means ± SEM (*n* = 6). Bars for each value with different letters were significantly different (*P* < 0.05).

As shown in Figure 3, apparent cholesterol absorption in rats was significantly depressed from −111.35 to −167.65% in RP-feeding as compared with CAS-feeding. As a result, apparent cholesterol absorption was more effectively inhibited by RP-E than RP-A (Figure 3).

## 4. Discussion

We examined the cholesterol-lowering potential of rice protein extracted by different method, suggesting that the hypocholesterolemic action of RP was associated with lower protein digestibility and higher fecal excretion of neutral sterols. Our findings clearly indicate that the digestibility of rice protein is a major factor to influence cholesterol metabolism through the inhibition of cholesterol absorption.

Conversion of cholesterol to bile acids is the principal regulated pathway whereby cholesterol is eliminated from the body, primarily via the enterohepatic circulation [7,8]. In the present study, our data suggested that the fecal excretion of bile acids can be affected by the digestibility of RP. Results indicated that RP-E significantly stimulated fecal excretion of bile acids (*P* < 0.05), whereas fecal bile acids excretion of RP-A was comparable to CAS (*P* > 0.05). These findings support the previous studies that alkaline treatment may improve the digestibility of rice protein [11,12], suggesting that RP-A appears to have less influence on binding bile acids in the intestinal tract due to its higher digestibility, in line with our previous findings [6]. Nevertheless, of interest was the finding that RP-A caused the cholesterol-lowering effect in growing rats under the present experimental condition, despite the fecal excretion of bile acids was not significantly stimulated by RP-A feeding. The precise mechanism by which RP affects cholesterol metabolism is not fully established, but the higher extent to which RP-E and RP-A enhanced excretions of neutral sterols in fecal output should be taken into account. Clearly, the finding observed in this study suggests that hypocholesterolemic action induced by RP cannot be explained by the result of a simple diversion of bile acids toward fecal excretion, whereas the inhibition of intestinal cholesterol absorption, which was induced by the lower digestibility, might be one of possible mechanisms exerted by rice protein due to its lower digestibility.

In this study, we provided the *in vivo* and *in vitro* evidences for the lower digestibility of RP as compared with CAS. These results confirmed the finding that the digestibility of RP-E extracted by α-amylase was significantly lower than RP-A by alkaline treatment, further supporting the view that the digestibility of RP could be affected by the processing of extraction [5,11,12]. More importantly, to support our hypothesis that lower digestibility of RP might produce the cholesterol-lowering action, we found a significant positive correlation between the protein digestibility and the concentration of plasma cholesterol (*r* = 0.7357, *P* < 0.01), as well as liver cholesterol accumulation (*r* = 0.9025, *P* < 0.01) (Table 7). These data clearly indicated that there was a close link of lower protein digestibility and cholesterol-lowering action exerted by RP.

The most frequently suggested mechanism responsible for the cholesterol-lowering effect of dietary protein is to inhibit the intestinal cholesterol absorption and an increase in fecal steroid excretion [5,16–18,24]. In the present study, our results confirm and expand this view, indicating that higher fecal excretions of neutral sterols and nitrogen were closely associated with lower cholesterol concentration in plasma and liver (Table 7). Results showed a significant negative correlation between fecal neutral sterols and plasma cholesterol concentration (*r* = −0.7451, *P* < 0.01), as well as liver cholesterol concentration (*r* = −0.8629, *P* < 0.01). Furthermore, with the increasing proportion of nitrogen in feces, the absorption of cholesterol was more efficaciously depressed in rats, indicating a significant positive correlation between the protein digestibility and the apparent cholesterol absorption (*r* = 0.8662, *P* < 0.01). Thus, with the lower digestibility, RP posses a cholesterol-lowering action through enhancing fecal excretion of neutral sterols, further suggesting the digestibility of rice protein is a major factor to influence cholesterol metabolism through the inhibition of cholesterol absorption.

Here, it must be noted that the hypocholesterolemic effect of RP-E was not more effective than that of RP-A. Also, we did not observe a difference of liver cholesterol-lowering effect between RP-E and RP-A. Thus, the question arises why the stronger inhibition of cholesterol absorption induced by RP-E with lower digestibility did not lead to lower cholesterol level in rats fed RP-E than those fed RP-A. The precise mechanism by which RP affects cholesterol metabolism is not fully understood, but the mechanism responsible for the effect on the removal of non-HDL-C from circulation by hepatic uptake should be taken into account. Our data obtained from plasma HDL-C and non-HDL-C indicated that RP-E and RP-A produced the similar ratio of non-HDL-C to HDL-C in the present study (RP-E, 0.42; RP-A, 0.45), which may result in the similar transfer of peripheral free cholesterol to the liver by a mechanism known as “reverse cholesterol transfer” promoted by HDL-C. Thus, the findings observed in this study suggest that, except for the inhibition of cholesterol absorption induced by lower digestibility of RP, other mechanisms, such as the stimulation of the uptake of lipoprotein cholesterol by the liver may also come into play to fully explain the hypocholesterolemic response to RP. Clearly, the observed effect of RP on cholesterol metabolism remains to be clarified in further studies.

Cholesterol absorption has been also suggested to be affected by amino acid composition. Vahouny *et al*. reported that the cholesterol-lowering effect of soy protein was due mainly to the ratio of arginine (Arg) to lysine (Lys), which was involved with the inhibition of intestinal lipid absorption [25]. Results shown by Vahouny *et al.* indicated that the addition of Arg to a CAS diet to increase the Arg/Lys ratio resulted in a slower rate of lipid absorption, and the addition of Lys to the soy protein diet to decrease the Arg/Lys ratio caused a faster rate of lipid absorption. In addition, some studies have led to better understanding of the higher ratio of Arg/Lys can result in an elevation in hepatic 7α-hydroxylase activity, which is a rate-limiting enzyme for conversion of cholesterol to bile acids [26]. Thus, whatever the mechanism of action, the effect of Arg/Lys ratio on cholesterol metabolism is important. Considering this view, the highest Arg/Lys ratio in RP-E (3.15), the higher in RP-A (2.56) and the lowest in CAS (0.44) might explain the different levels in plasma cholesterol and fecal output of sterols in the present study. In light of these findings, the higher ratio of Arg/Lys in RP, which may depress the cholesterol absorption to result in hypocholesterolemia, should be also emphasized in the present study. Thus, in respect to the influence on digestibility, the difference of amino acid composition induced by the different extraction method may represent a principal mechanism for inhibition of cholesterol absorption to induce the cholesterol-lowering action of RP.

Additionally, some studies have suggested that other non-protein contents, e.g., carbohydrate, dietary fiber, unsaturated fatty acids, also could induce the cholesterol-lowering action [27]. However, it does not seem very likely that the other remaining non-protein components, e.g., dietary fiber, carbohydrate, in RP may contribute to the cholesterol-lowering effects in this study because their concentrations are extremely low compared to the concentrations normally used for the induction of hypolipidemia. This may explain the phenomenon that RP-E containing more dietary fiber could not produce more effective cholesterol-lowering effect than RP-A. Clearly, additional studies are required to confirm this view.

## 5. Conclusions

The present study demonstrates that digestibility of rice protein plays a major role in the modification of cholesterol metabolism. The hypocholesterolemic action of rice protein is attributed to the enhanced fecal excretion of neutral sterols, varying with the digestibility of rice protein. Our results suggest that the inhibition of cholesterol absorption, which is closely associated with the digestibility and the ratio of Arg/Lys, may be the main modulator responsible for the cholesterol-lowering action of rice protein. The precise mechanisms involved in the hypocholesterolemic responses to rice protein await more detailed investigation.

## Figures and Tables

**Table 1:** Composition of experimental diets (g/kg diet).

Ingredients	CAS	RP-A	RP-E
CAS a	229.1	−	−
RP-A b	−	231.5	−
RP-E c	−	−	251.3
Sucrose	100.0	100.0	100.0
Cellulose	50.0	50.0	50.0
Soybean oil	70.0	70.0	70.0
β-cornstarch	500.4	501.0	481.2
Mineral mix d	35.0	35.0	35.0
Vitamin mix e	10.0	10.0	10.0
Choline bitartrate	2.5	2.5	2.5
Tert-butylhydroquinone	0.014	0.014	0.014
l-Cystine	3.0	−	−

a CAS, casein; protein concentration 873 g/kg, obtained from Hualing Industrial Group (Gansu, China);

b RP-A, rice protein extracted by alkaline treatment; protein concentration 864 g/kg, prepared by our laboratory;

c RP-E, rice protein extracted by α-amylase; protein concentration 796 g/kg, prepared by our laboratory;

d Mineral mix, AIN-93G-MX (Nosan Corp., Yokohama, Japan);

e Vitamin mix, AIN-93-VX (Nosan Corp., Yokohama, Japan).

**Table 2:** Composition of dietary proteins (%).

Composition	CAS	RP-A	RP-E
Moisture	9.7	9.8	10.9
Protein	87.3	86.4	79.6
Carbohydrate	0.2	0.9	2.5
Lipids	0.5	0.8	0.8
Ash	1.7	1.0	1.5
Dietary fiber	0.6	1.1	4.7

Data are averages of triplicate analysis. CAS, casein; RP-A, rice protein extracted by alkaline treatment; RP-E, rice protein extracted by α-amylase.

**Table 3:** Amino acid composition of dietary protein (μg/mg).

Amino acid	CAS	RP-A	RP-E
Asp	67.3	87.7	89.7
Thr	40.7	29.4	30.7
Ser	50.9	48.7	47.9
Glu	163.6	192.3	189.0
Gly	19.5	44.2	44.0
Ala	33.9	55.5	53.9
Val	58.4	63.6	60.7
Ile	49.6	41.2	43.2
Leu	84.5	81.4	83.6
Met	29.9	21.2	17.7
Cys	2.6	21.8	20.8
Tyr	55.5	56.4	46.9
Phe	50.9	51.9	51.3
Lys	75.8	34.3	27.9
Arg	33.3	87.8	88.0
His	29.9	24.3	23.8
Pro	94.6	37.6	37.4

Data are averages of triplicate analysis.

**Table 4:** Body weight gain, food intake and plasma lipids in rats.

Parameters	CAS	RP-A	RP-E
Body weight gain (g/day)	5.56 ± 0.21 a	4.61 ± 0.15 b	4.45 ± 0.16 b
Food intake (g/day)	20.04 ± 0.64	19.02 ± 0.29	18.88 ± 0.42
Plasma Cholesterol (mmol/L)			
TC	1.44 ± 0.04 a	1.26 ± 0.05 b	1.21 ± 0.04 b
VLDL-C	0.17 ± 0.01 a	0.13 ± 0.01 b	0.12 ± 0.01 b
LDL-C	0.39 ± 0.06 a	0.26 ± 0.05 b	0.24 ± 0.06 b
HDL-C	0.88 ± 0.04	0.87 ± 0.02	0.85 ± 0.04
Non-HDL/HDL-C (mol/mol)	0.64 ± 0.07 a	0.45 ± 0.06 b	0.42 ± 0.05 b
Triglyceride (mmol/L)	0.38 ± 0.02 a	0.35 ± 0.01 ab	0.33 ± 0.01 b
TG/HDL-C (mol/mol)	0.43 ± 0.03	0.40 ± 0.01	0.39 ± 0.01

Values are means ± SEM for six rats.

a,b Values with different letters are significant different, *P* < 0.05.

**Table 5:** Hepatic lipids and deposit fat of rats fed experimental diets.

Parameters	CAS	RP-A	RP-E
Liver			
Liver weight (g)	8.80 ± 0.18 a	7.98 ± 0.31 b	7.65 ± 0.28 b
Total lipids (mg/g liver)	125.71 ± 11.34 a	94.09 ± 6.28 b	88.98 ± 2.19 b
Cholesterol (μmol/g liver)			
total	14.90 ± 0.53 a	11.17 ± 0.36 b	10.29 ± 0.31 b
free	11.00 ± 0.39 a	8.97 ± 0.29 b	8.59 ± 0.27 b
esterified	3.90 ± 0.14 a	2.20 ± 0.07 b	1.70 ± 0.04 b
esterification ratio (%)	26.17 ± 1.57 a	19.70 ± 0.87 b	16.52 ± 0.59 b
Triglyceride (μmol/g liver)	63.49 ± 2.66 a	41.05 ± 1.37 b	39.12 ± 0.68 b
Deposit fat (g/kg body weight)			
perirenal	14.49 ± 0.49 a	13.11 ± 0.43 ab	12.72 ± 0.25 b
epididymal	10.93 ± 0.37 a	9.89 ± 0.32 ab	9.59 ± 0.19 b

Values are means ± SEM for six rats.

a,b Values with different letters are significant different, *P* < 0.05.

**Table 6:** Fecal excretion of steroid and nitrogen in rats fed experimental diets.

Fecal excretion	CAS	RP-A	RP-E
Fecal output (g dry weight/3 day)	4.68 ± 0.11 b	4.81 ± 0.15 ab	5.10 ± 0.17 a
Bile acids (μmol/3 day)	114.62 ± 5.12 b	131.21 ± 5.95 b	154.86 ± 8.65 a
Neutral sterol (μmol/3 day)			
total	58.26 ± 2.52 c	123.13 ± 3.85 b	155.93 ± 6.60 a
cholesterol	23.28 ± 1.09 c	32.21 ± 1.70 b	38.33 ± 1.90 a
coprostanol	20.34 ± 1.55 c	48.85 ± 1.97 b	62.76 ± 3.46 a
other sterols	14.64 ± 1.06 c	42.07 ± 1.88 b	54.84 ± 2.42 a
Triglyceride (μmol/3 day)	7.44 ± 0.38 b	9.55 ± 0.57 a	10.82 ± 0.55 a
Nitrogen (mg/3 day)	88.27 ± 0.81 c	220.21 ± 4.72 b	280.78 ± 3.79 a
Fecal protein (g/3 day)	0.55 ± 0.01 c	1.38 ± 0.03 b	1.75 ± 0.02 a
Intake of dietary protein (g/3 day)	9.62 ± 0.31	9.13 ± 0.14	9.06 ± 0.20
Apparent protein digestibility (%)	94.28 ± 0.20 a	84.88 ± 0.47 b	80.68 ± 0.61 c

Values are means ± SEM for six rats.

a-c Values with different letters are significant different, *P* < 0.05.

**Table 7:** Correlation analyses of factors to affect plasma and liver cholesterol concentrations in rats fed dietary proteins.

Variable		Slope	Intercept	Correlation Coefficient
*X* (independent)	*Y* (Dependent)			
Fecal nitrogen excretion	Plasma cholesterol	−0.0012	1.547	0.7586 *
Fecal neutral sterol excretion	Plasma cholesterol	−0.0024	1.568	0.7451 *
Apparent protein digestibility	Plasma cholesterol	0.0168	0.151	0.7357 *
Fecal nitrogen excretion	Liver cholesterol	−0.0247	16.961	0.9154 *
Fecal neutral sterol excretion	Liver cholesterol	−0.0450	17.177	0.8629 *
Apparent protein digestibility	Liver cholesterol	0.3396	17.285	0.9025 *

* *P* < 0.01.
